# Endovascular Retrieval of a Peeled-Off Fragment of a Hydrophilic Guidewire Embolized Into the Pulmonary Artery Using a Unique Loop-Snare Torque-Entanglement Technique

**DOI:** 10.7759/cureus.89725

**Published:** 2025-08-10

**Authors:** Krishna Prasad Bellam Premnath, Mohd Shariq, Shady Hegab, Sana

**Affiliations:** 1 Interventional Radiology, Queen's Hospital, Romford, GBR; 2 Clinical Radiology, Queen's Hospital, Romford, GBR

**Keywords:** hydrophilic wire, intravascular foreign body, loop snare, pulmonary artery embolization, retained guidewire

## Abstract

Pulmonary vascular embolization of the stripped coating of a hydrophilic guidewire is a rare complication. Endovascular retrieval is preferred over surgical treatment or conservative management. Most of the commonly used endovascular retrieval techniques fall short when the foreign body does not present a free edge to grab. There are a few innovative techniques that can be applied to retrieve such difficult foreign bodies. This case report describes a unique loop-snare torque-entanglement technique for the retrieval of such a foreign body out of the pulmonary vasculature.

## Introduction

Hydrophilic wires are used extensively in endovascular procedures because of reduced friction, improved maneuverability, enhanced safety, lesion crossability, and lesser thrombogenicity. The most widely used hydrophilic guidewire is the Terumo Glidewire (Terumo Corporation, Tokyo, Japan) [1]. They have two major disadvantages: since they can cross lesions easily, once in a false passage, they can track for long distances without providing resistance tactile feedback, and their hydrophilic coat can strip off from the rest of the wire when inadvertent shearing forces are applied, notoriously seen while pulling the wire forcefully across the beveled end of a needle [2-5]. If this is identified early before complete detachment of the stripped coating, the beveled needle-guidewire combination can be pulled out together, avoiding an intravascular foreign body. There are different methods of retrieval of embolized hydrophilic-coated foreign bodies from blood vessels [2-5]. The most used loop snares to retrieve prove ineffective if the linear foreign body does not present a free edge or is lodged in a small vessel [5-7]. We present a unique technique for removing such a foreign body from a pulmonary artery branch using a loop-snare torque-entanglement technique.

## Case presentation

**Figure 1:**
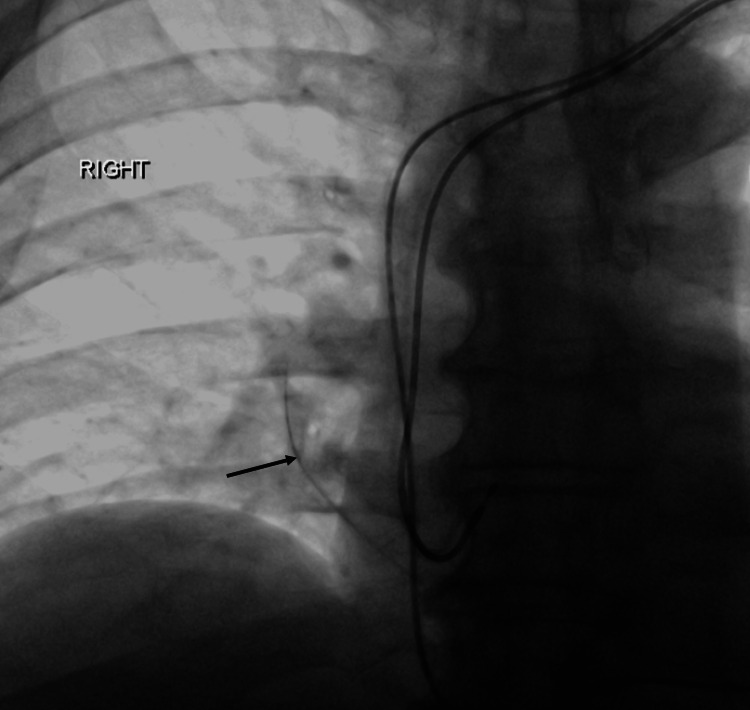
Fluoroscopic image post right ventricular lead replacement Fluoroscopic image shows a linear radiodensity in the right lung medial aspect lower zone (black arrow) representing the embolized hydrophilic coating of the guidewire.

A 66-year-old gentleman was noted to have a possible loss of the atrial lead of his dual-chamber pacemaker in his post-procedure X-ray. Due to intermittent loss of ventricular capture with pacing showing high ventricular lead threshold, he underwent a lead revision and right ventricular lead replacement successfully. During the procedure, a thin radio-opaque shadow was noted in the middle-lower zone of the right lung (Figure 1), and a hydrophilic coating defect in the Terumo Glidewire was seen ex vivo.

The cardiologist performing the procedure recalled difficulty in removing a Terumo Glidewire out through a beveled metallic access needle. It was concluded that the shadow was an embolized, sheared-off hydrophilic coating lodged into the right pulmonary artery vasculature. The patient was referred to the on-call interventional radiology team for an attempt at retrieval of the foreign body. The patient did not demonstrate any symptoms that could be attributed to the embolized guidewire hydrophilic coating fragment.

**Figure 2:**
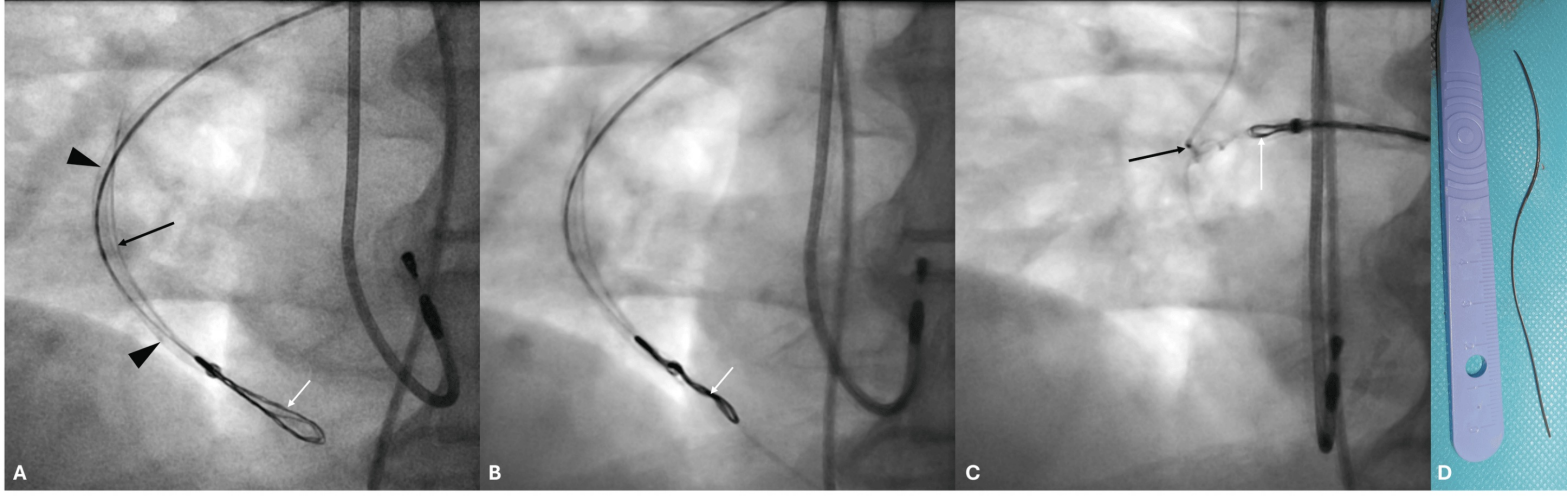
Intraprocedural fluoroscopic images during retrieval of the embolized pulmonary foreign body Intraprocedural fluoroscopic images (A, B, and C). Image A shows the snare placed alongside the foreign body (A), with the black arrow pointing to the foreign body, black arrowheads indicating the snare delivery catheter, and the white arrow pointing to the nitinol loops of the snare. Image B shows the twisted snare entangling with the foreign body (B), with the white arrow pointing to the snare, which was twisted/torqued to intertwine it with the foreign body. Image C shows the retrieval of the snare and entangled foreign body (C), where the white arrow points to the snare and the black arrow to the entwined foreign body. Image D shows the retrieved foreign body adjacent to the graduated handle of a blade for scale.

The patient was moved to the interventional radiology theater. The right common femoral venous retrograde access was obtained, and a short 6F vascular sheath was placed. The right lower lobe pulmonary artery and further the medial branch harboring the foreign body were cannulated using a combination of a 5F angled vertebral catheter (Cordis Corporation, Miami Lakes, FL) and an angled tip Terumo Glidewire, with the help of different directional (left and right anterior oblique) projections. An Amplatz Extra Stiff Guide wire (Cook Medical, Bloomington, IN) with a straight 1 cm floppy tip was subsequently placed into the branch through the angled vertebral catheter, and the snare catheter of a 6F Trefoil En-Snare (Merit Medical Systems, Inc., South Jordan, UT) was navigated over the wire into the pulmonary artery branch. Neither end of the foreign body could be snared since the ends were closely abutting the vessel wall, and the branch of the pulmonary artery was small. The snare was subsequently placed alongside the foreign body and was torque-rotated multiple times, resulting in entanglement of the foreign body with the snare. The snare was partially withdrawn till resistance was felt, and the snare and entangled foreign body were retrieved as a single unit (Figure 2).

The retrieved foreign body was black in color, measured 8.2 cm, and resembled the coating of the Terumo Glidewire. A post-retrieval fluoroscopic image confirmed complete successful retrieval of the foreign body. The patient remained hemodynamically stable during the procedure and tolerated the procedure well under local anesthesia. The patient did not develop any complications and was discharged uneventfully, and was advised to follow up with his cardiologist.

## Discussion

Embolization of the coating of a hydrophilic guidewire, like the Terumo Glidewire, is a rare but significant complication that can happen during interventional vascular procedures. Hydrophilic coating provides flexibility and trackability of the wire, especially for navigating through challenging anatomy and crossing difficult occlusions. Delamination of hydrophilic coating and ensuing embolization can occur with prolonged manipulation, excessive torque, or excessive traction across severely angulated vessels, across the beveled tip of a metallic needle, or across a metallic stent, especially when jailed between the stent and vessel wall [1,3-6,8]. Careful handling of hydrophilic guidewires, knowledge, and early recognition of potential adverse events are important to avoid these complications. Extreme attentiveness is needed while handling a hydrophilic wire through a metallic access needle, especially when the needle is beveled. Using a plastic introducer like an 18-G intravenous cannula instead of a beveled metallic needle or using a non-hydrophilic metallic wire would avoid this potential complication.

The clinical presentation of embolized foreign bodies in the pulmonary artery can range from being asymptomatic to causing hemodynamic instability. These may sometimes go unnoticed and be detected later incidentally on radiography [5]. It is best to remove these foreign bodies when possible, because of the potential risk of infection, septicemia, thrombosis, or lung erosion and hemorrhage [5]. Percutaneous retrieval is regarded as the standard treatment for such intravascular foreign bodies. Different techniques and devices have been described for the retrieval of such a linear foreign body from the vasculature, both systemic and pulmonary, which include loop snares, shaped guidewires, Fogarty balloon catheters, alligator forceps, Dormia baskets, etc. [2, 5-7]. The choice of retrieval equipment depends on multiple factors, like the size, shape, and location of the embolized material. Loop snares are the most commonly used retrieval device. Loop snares, however, can be ineffective when the embolized foreign body does not present a free end for engaging within the loop of the snare for grabbing. Different techniques have been described for the retrieval of such foreign bodies without a free edge, which include either repositioning the foreign body using a hook device like a pigtail catheter so that a free end is available for snaring [5] or entangling the foreign body with multiple guidewires with a manually made spiral-shaped tip [6,7]. Using a single wire is unlikely to be successful, and multiple wires provide more stability and increase the chances of entangling and retrieval [6]. Alligator forceps might be an alternative option, but inadvertent inclusion of the vessel wall might result in vessel injury and significant bleeding.

## Conclusions

The technique described in this case report is a yet undescribed method of dealing with such difficult-to-retrieve foreign bodies. This can be compared with retrieval using multiple guidewires described earlier, with the different segments of multiple loops of the snare acting like multiple guidewires, but entanglement is achieved by torquing the snare instead of pre-shaping the tip of the guidewires. This technique is safe since there is no direct vessel wall injury, and safe entanglement without significant vessel distortion can be achieved.
